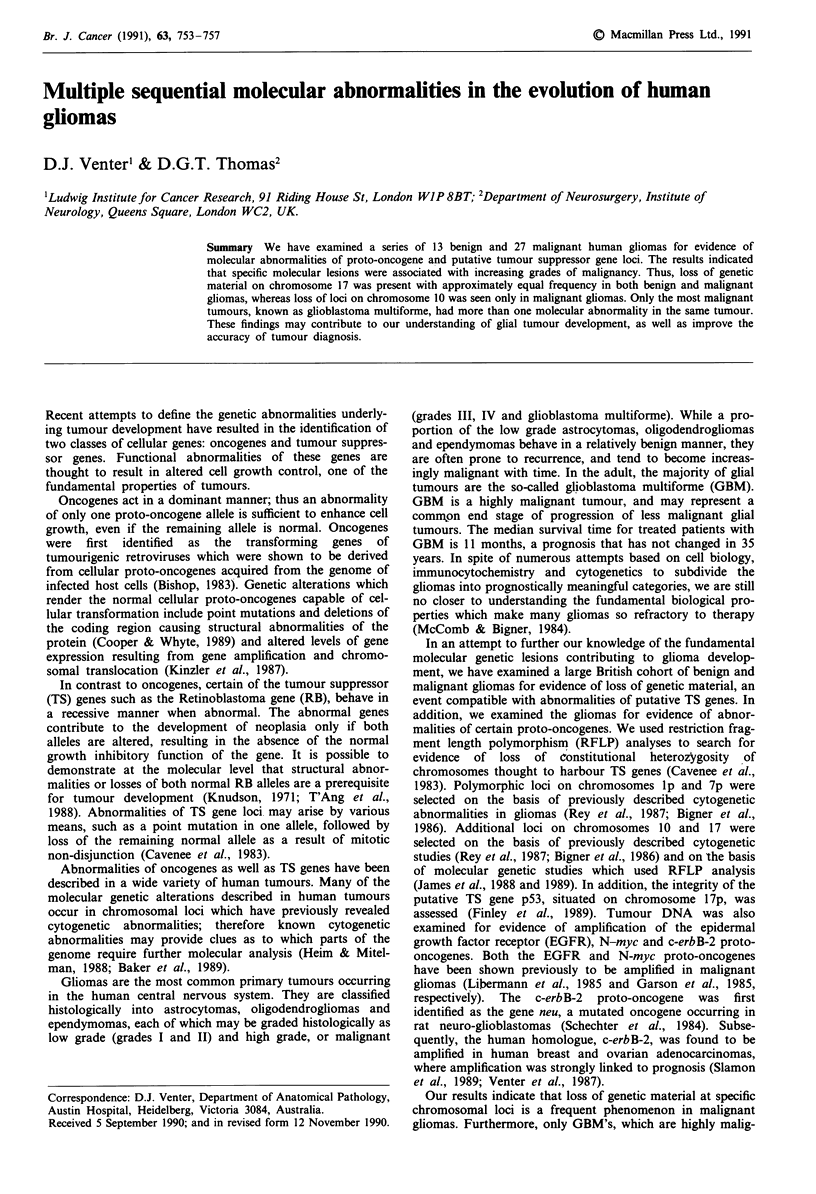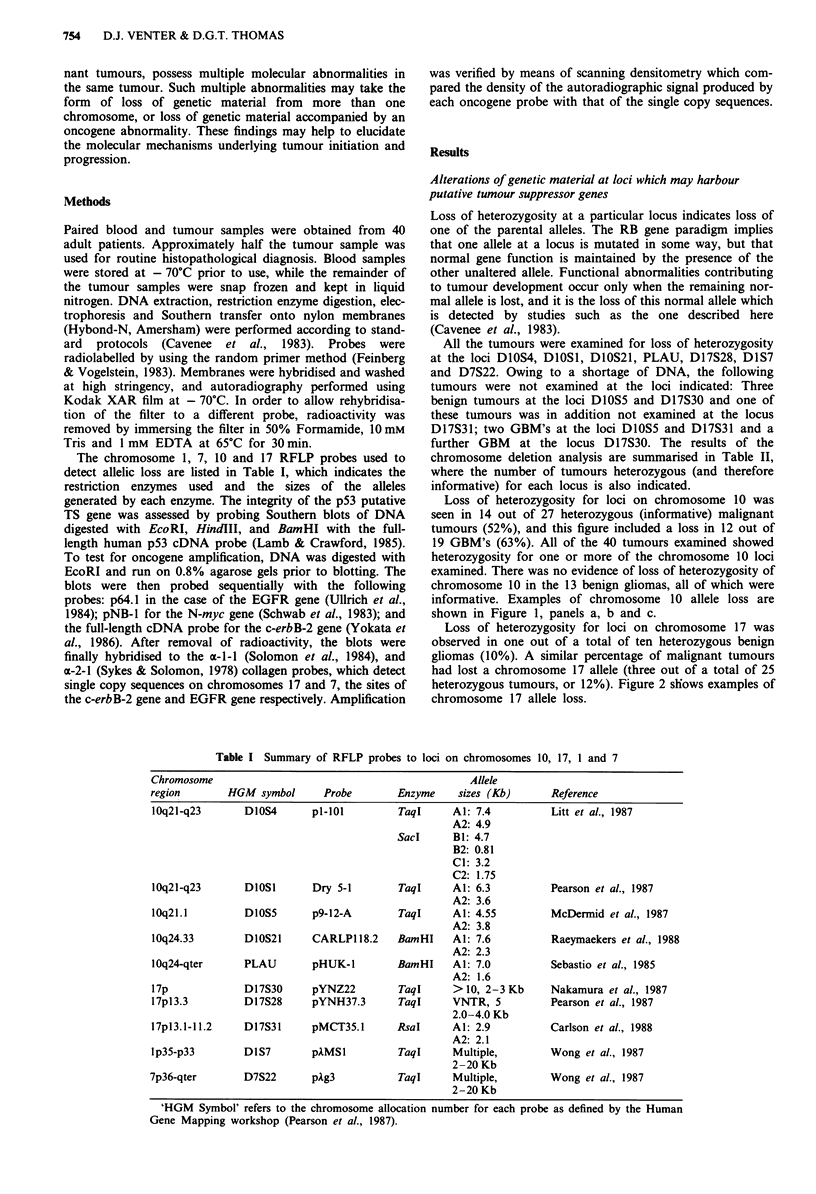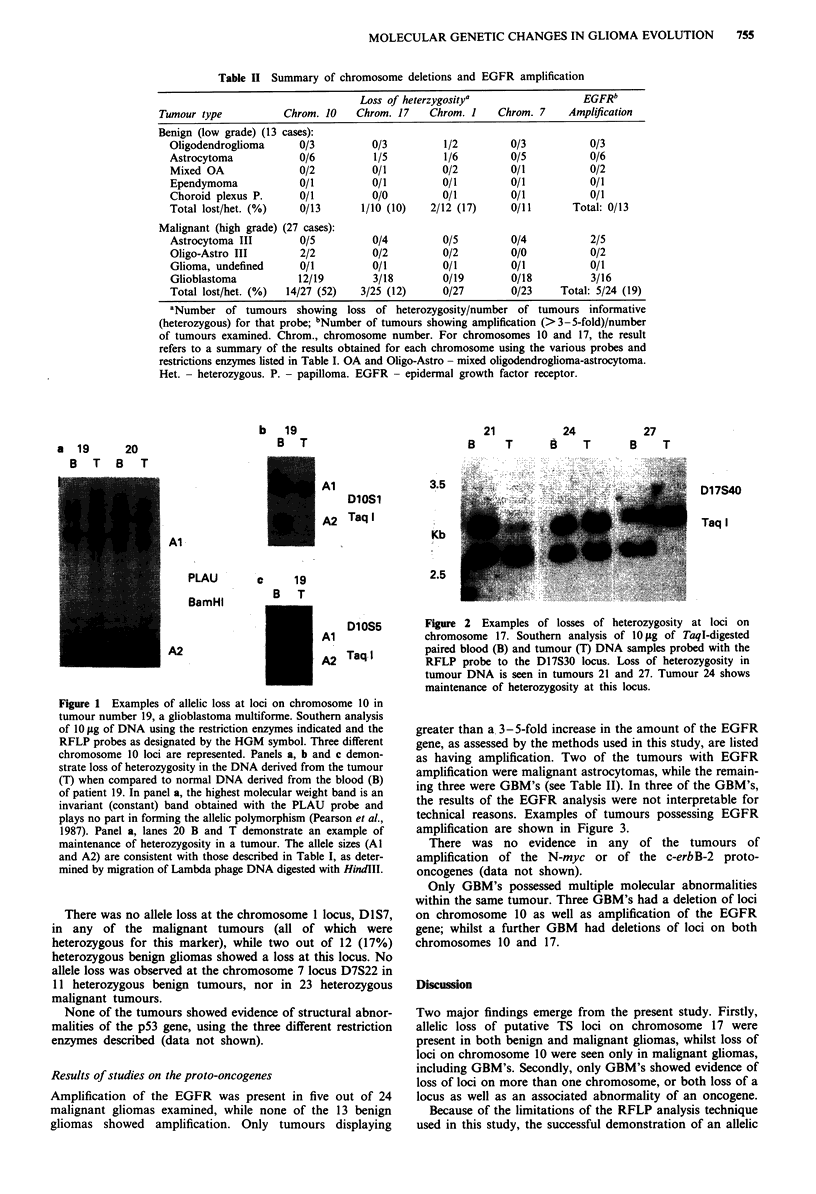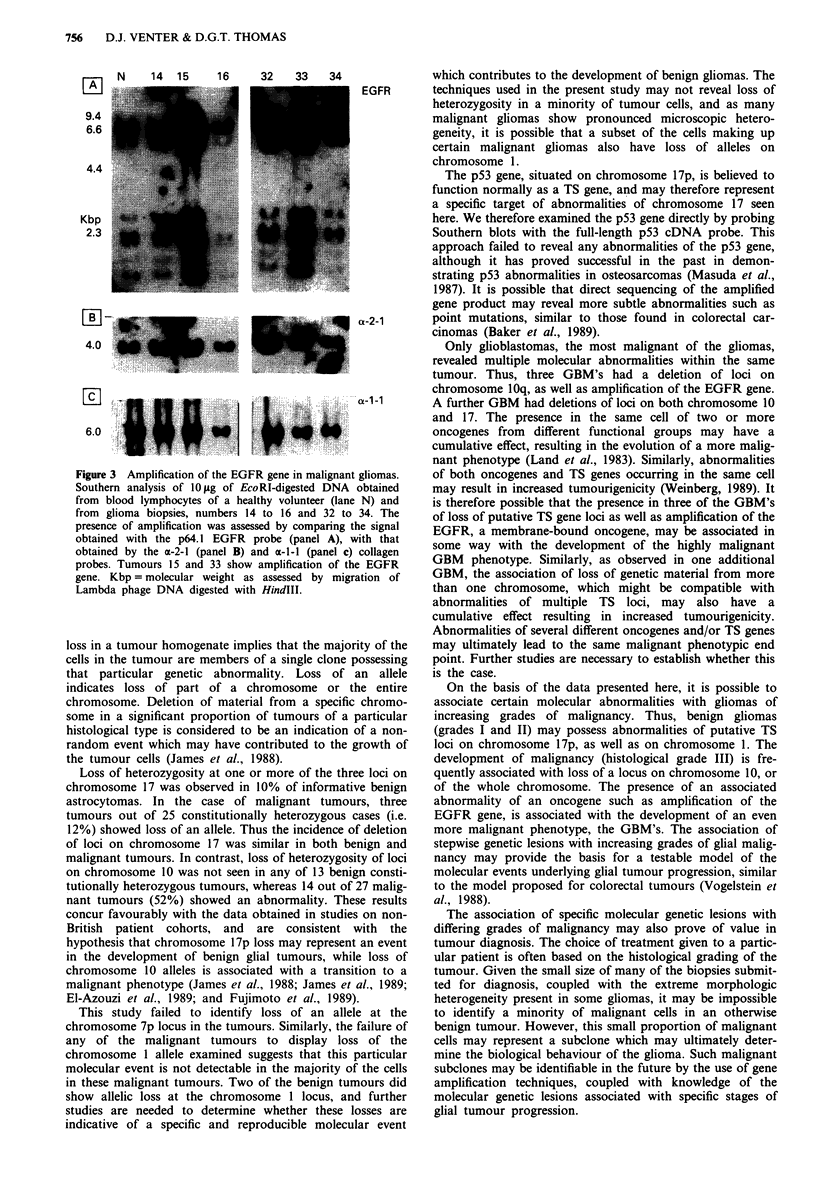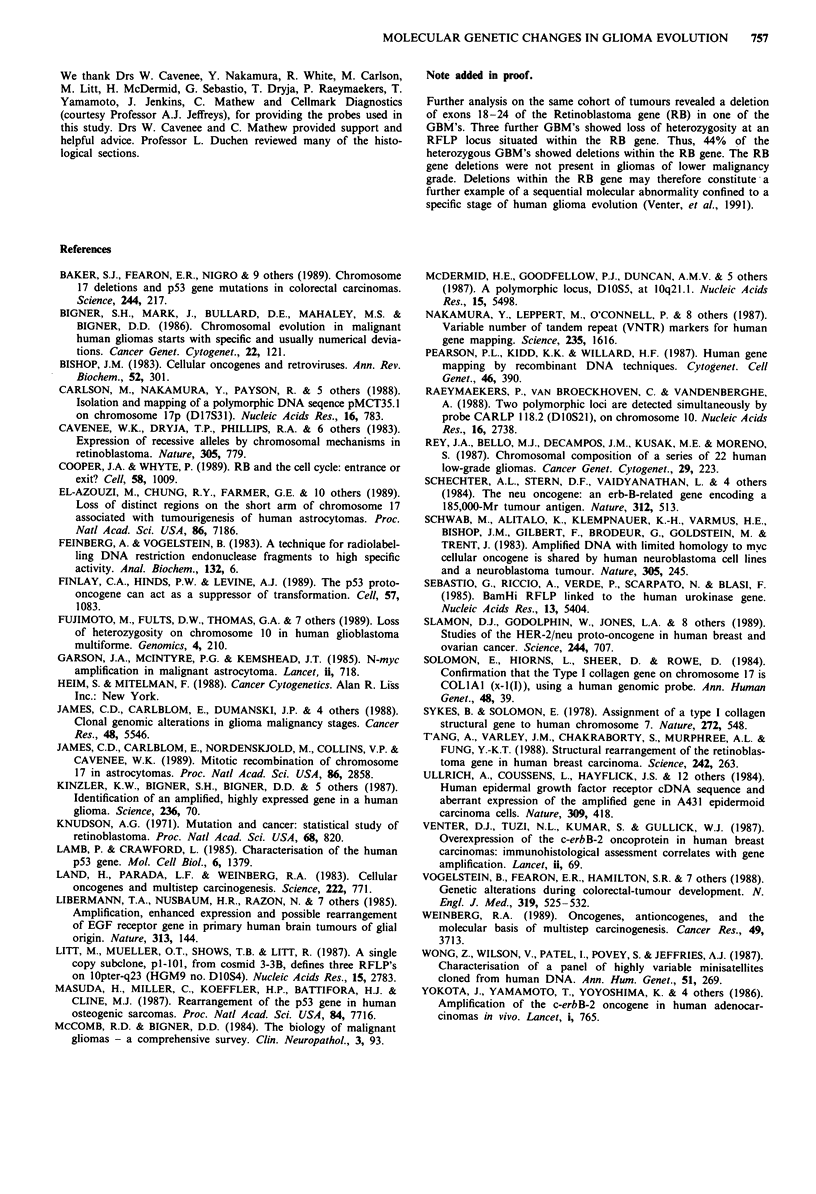# Multiple sequential molecular abnormalities in the evolution of human gliomas.

**DOI:** 10.1038/bjc.1991.168

**Published:** 1991-05

**Authors:** D. J. Venter, D. G. Thomas

**Affiliations:** Ludwig Institute for Cancer Research, London, UK.

## Abstract

**Images:**


Br. J. Cancer (1991), 63, 753-757 © Macmillan Press Ltd., 1991

### Multiple sequential molecular abnormalities in the evolution of human gliomas

D.J. Venter[1] & D.G.T. Thomas[2]

[1]*Ludwig Institute for Cancer Research, 91 Riding House St, London W1P 8BT;* [2]*Department of Neurosurgery, Institute of Neurology, Queens Square, London WC2, UK.*

**Summary** We have examined a series of 13 benign and 27 malignant human gliomas for evidence of molecular abnormalities of proto-oncogene and putative tumour suppressor gene loci. The results indicated that specific molecular lesions were associated with increasing grades of malignancy. Thus, loss of genetic material on chromosome 17 was present with approximately equal frequency in both benign and malignant gliomas, whereas loss of loci on chromosome 10 was seen only in malignant gliomas. Only the most malignant tumours, known as glioblastoma multiforme, had more than one molecular abnormality in the same tumour. These findings may contribute to our understanding of glial tumour development, as well as improve the accuracy of tumour diagnosis.

Recent attempts to define the genetic abnormalities underlying tumour development have resulted in the identification of two classes of cellular genes: oncogenes and tumour suppressor genes. Functional abnormalities of these genes are thought to result in altered cell growth control, one of the fundamental properties of tumours.

Oncogenes act in a dominant manner; thus an abnormality of only one proto-oncogene allele is sufficient to enhance cell growth, even if the remaining allele is normal. Oncogenes were first identified as the transforming genes of tumourigenic retroviruses which were shown to be derived from cellular proto-oncogenes acquired from the genome of infected host cells (Bishop, 1983). Genetic alterations which render the normal cellular proto-oncogenes capable of cellular transformation include point mutations and deletions of the coding region causing structural abnormalities of the protein (Cooper & Whyte, 1989) and altered levels of gene expression resulting from gene amplification and chromosomal translocation (Kinzler *et al.*, 1987).

In contrast to oncogenes, certain of the tumour suppressor (TS) genes such as the Retinoblastoma gene (RB), behave in a recessive manner when abnormal. The abnormal genes contribute to the development of neoplasia only if both alleles are altered, resulting in the absence of the normal growth inhibitory function of the gene. It is possible to demonstrate at the molecular level that structural abnormalities or losses of both normal RB alleles are a prerequisite for tumour development (Knudson, 1971; T'Ang *et al.*, 1988). Abnormalities of TS gene loci may arise by various means, such as a point mutation in one allele, followed by loss of the remaining normal allele as a result of mitotic non-disjunction (Cavenee *et al.*, 1983).

Abnormalities of oncogenes as well as TS genes have been described in a wide variety of human tumours. Many of the molecular genetic alterations described in human tumours occur in chromosomal loci which have previously revealed cytogenetic abnormalities; therefore known cytogenetic abnormalities may provide clues as to which parts of the genome require further molecular analysis (Heim & Mitelman, 1988; Baker *et al.*, 1989).

Gliomas are the most common primary tumours occurring in the human central nervous system. They are classified histologically into astrocytomas, oligodendrogliomas and ependymomas, each of which may be graded histologically as low grade (grades I and II) and high grade, or malignant (grades III, IV and glioblastoma multiforme). While a proportion of the low grade astrocytomas, oligodendrogliomas and ependymomas behave in a relatively benign manner, they are often prone to recurrence, and tend to become increasingly malignant with time. In the adult, the majority of glial tumours are the so-called glioblastoma multiforme (GBM). GBM is a highly malignant tumour, and may represent a common end stage of progression of less malignant glial tumours. The median survival time for treated patients with GBM is 11 months, a prognosis that has not changed in 35 years. In spite of numerous attempts based on cell biology, immunocytochemistry and cytogenetics to subdivide the gliomas into prognostically meaningful categories, we are still no closer to understanding the fundamental biological properties which make many gliomas so refractory to therapy (McComb & Bigner, 1984).

In an attempt to further our knowledge of the fundamental molecular genetic lesions contributing to glioma development, we have examined a large British cohort of benign and malignant gliomas for evidence of loss of genetic material, an event compatible with abnormalities of putative TS genes. In addition, we examined the gliomas for evidence of abnormalities of certain proto-oncogenes. We used restriction fragment length polymorphism (RFLP) analyses to search for evidence of loss of constitutional heterozygosity of chromosomes thought to harbour TS genes (Cavenee *et al.*, 1983). Polymorphic loci on chromosomes 1p and 7p were selected on the basis of previously described cytogenetic abnormalities in gliomas (Rey *et al.*, 1987; Bigner *et al.*, 1986). Additional loci on chromosomes 10 and 17 were selected on the basis of previously described cytogenetic studies (Rey *et al.*, 1987; Bigner *et al.*, 1986) and on the basis of molecular genetic studies which used RFLP analysis (James *et al.*, 1988 and 1989). In addition, the integrity of the putative TS gene p53, situated on chromosome 17p, was assessed (Finley *et al.*, 1989). Tumour DNA was also examined for evidence of amplification of the epidermal growth factor receptor (EGFR), N-*myc* and c-*erb*B-2 proto-oncogenes. Both the EGFR and N-*myc* proto-oncogenes have been shown previously to be amplified in malignant gliomas (Libermann *et al.*, 1985 and Garson *et al.*, 1985, respectively). The c-*erb*B-2 proto-oncogene was first identified as the gene *neu*, a mutated oncogene occurring in rat neuro-glioblastomas (Schechter *et al.*, 1984). Subsequently, the human homologue, c-*erb*B-2, was found to be amplified in human breast and ovarian adenocarcinomas, where amplification was strongly linked to prognosis (Slamon *et al.*, 1989; Venter *et al.*, 1987).

Our results indicate that loss of genetic material at specific chromosomal loci is a frequent phenomenon in malignant gliomas. Furthermore, only GBM's, which are highly malig-

Correspondence: D.J. Venter, Department of Anatomical Pathology, Austin Hospital, Heidelberg, Victoria 3084, Australia.
Received 5 September 1990; and in revised form 12 November 1990.

nant tumours, possess multiple molecular abnormalities in the same tumour. Such multiple abnormalities may take the form of loss of genetic material from more than one chromosome, or loss of genetic material accompanied by an oncogene abnormality. These findings may help to elucidate the molecular mechanisms underlying tumour initiation and progression.

### Methods

Paired blood and tumour samples were obtained from 40 adult patients. Approximately half the tumour sample was used for routine histopathological diagnosis. Blood samples were stored at − 70°C prior to use, while the remainder of the tumour samples were snap frozen and kept in liquid nitrogen. DNA extraction, restriction enzyme digestion, electrophoresis and Southern transfer onto nylon membranes (Hybond-N, Amersham) were performed according to standard protocols (Cavenee *et al.*, 1983). Probes were radiolabelled by using the random primer method (Feinberg & Vogelstein, 1983). Membranes were hybridised and washed at high stringency, and autoradiography performed using Kodak XAR film at − 70°C. In order to allow rehybridisation of the filter to a different probe, radioactivity was removed by immersing the filter in 50% Formamide, 10 mM Tris and 1 mM EDTA at 65°C for 30 min.

The chromosome 1, 7, 10 and 17 RFLP probes used to detect allelic loss are listed in Table I, which indicates the restriction enzymes used and the sizes of the alleles generated by each enzyme. The integrity of the p53 putative TS gene was assessed by probing Southern blots of DNA digested with *Eco*RI, *Hind*III, and *Bam*HI with the full-length human p53 cDNA probe (Lamb & Crawford, 1985). To test for oncogene amplification, DNA was digested with EcoRI and run on 0.8% agarose gels prior to blotting. The blots were then probed sequentially with the following probes: p64.1 in the case of the EGFR gene (Ullrich *et al.*, 1984); pNB-1 for the N-*myc* gene (Schwab *et al.*, 1983); and the full-length cDNA probe for the c-*erb*B-2 gene (Yokata *et al.*, 1986). After removal of radioactivity, the blots were finally hybridised to the α-1-1 (Solomon *et al.*, 1984), and α-2-1 (Sykes & Solomon, 1978) collagen probes, which detect single copy sequences on chromosomes 17 and 7, the sites of the c-*erb*B-2 gene and EGFR gene respectively. Amplification was verified by means of scanning densitometry which compared the density of the autoradiographic signal produced by each oncogene probe with that of the single copy sequences.

### Results

### *Alterations of genetic material at loci which may harbour putative tumour suppressor genes*

Loss of heterozygosity at a particular locus indicates loss of one of the parental alleles. The RB gene paradigm implies that one allele at a locus is mutated in some way, but that normal gene function is maintained by the presence of the other unaltered allele. Functional abnormalities contributing to tumour development occur only when the remaining normal allele is lost, and it is the loss of this normal allele which is detected by studies such as the one described here (Cavenee *et al.*, 1983).

All the tumours were examined for loss of heterozygosity at the loci D10S4, D10S1, D10S21, PLAU, D17S28, D1S7 and D7S22. Owing to a shortage of DNA, the following tumours were not examined at the loci indicated: Three benign tumours at the loci D10S5 and D17S30 and one of these tumours was in addition not examined at the locus D17S31; two GBM's at the loci D10S5 and D17S31 and a further GBM at the locus D17S30. The results of the chromosome deletion analysis are summarised in Table II, where the number of tumours heterozygous (and therefore informative) for each locus is also indicated.

Loss of heterozygosity for loci on chromosome 10 was seen in 14 out of 27 heterozygous (informative) malignant tumours (52%), and this figure included a loss in 12 out of 19 GBM's (63%). All of the 40 tumours examined showed heterozygosity for one or more of the chromosome 10 loci examined. There was no evidence of loss of heterozygosity of chromosome 10 in the 13 benign gliomas, all of which were informative. Examples of chromosome 10 allele loss are shown in Figure 1, panels a, b and c.

Loss of heterozygosity for loci on chromosome 17 was observed in one out of a total of ten heterozygous benign gliomas (10%). A similar percentage of malignant tumours had lost a chromosome 17 allele (three out of a total of 25 heterozygous tumours, or 12%). Figure 2 shows examples of chromosome 17 allele loss.

**Table I** Summary of RFLP probes to loci on chromosomes 10, 17, 1 and 7

| *Chromosome region* | *HGM symbol* | *Probe* | *Enzyme* | *Allele sizes (Kb)* | *Reference* |
|---|---|---|---|---|---|
| 10q21-q23 | D10S4 | p1-101 | *Taq*I | A1: 7.4<br>A2: 4.9 | Litt *et al.*, 1987 |
| | | | *Sac*I | B1: 4.7<br>B2: 0.81<br>C1: 3.2<br>C2: 1.75 | |
| 10q21-q23 | D10S1 | Dry 5-1 | *Taq*I | A1: 6.3<br>A2: 3.6 | Pearson *et al.*, 1987 |
| 10q21.1 | D10S5 | p9-12-A | *Taq*I | A1: 4.55<br>A2: 3.8 | McDermid *et al.*, 1987 |
| 10q24.33 | D10S21 | CARLP118.2 | *Bam*HI | A1: 7.6<br>A2: 2.3 | Raeymaekers *et al.*, 1988 |
| 10q24-qter | PLAU | pHUK-1 | *Bam*HI | A1: 7.0<br>A2: 1.6 | Sebastio *et al.*, 1985 |
| 17p | D17S30 | pYNZ22 | *Taq*I | >10, 2–3 Kb | Nakamura *et al.*, 1987 |
| 17p13.3 | D17S28 | pYNH37.3 | *Taq*I | VNTR, 5<br>2.0–4.0 Kb | Pearson *et al.*, 1987 |
| 17p13.1-11.2 | D17S31 | pMCT35.1 | *Rsa*I | A1: 2.9<br>A2: 2.1 | Carlson *et al.*, 1988 |
| 1p35-p33 | D1S7 | pλMS1 | *Taq*I | Multiple,<br>2–20 Kb | Wong *et al.*, 1987 |
| 7p36-qter | D7S22 | pλg3 | *Taq*I | Multiple,<br>2–20 Kb | Wong *et al.*, 1987 |

'HGM Symbol' refers to the chromosome allocation number for each probe as defined by the Human Gene Mapping workshop (Pearson *et al.*, 1987).

**Table II** Summary of chromosome deletions and EGFR amplification

| Tumour type | Chrom. 10 | *Loss of heterozygosity*[a] Chrom. 17 | Chrom. 1 | Chrom. 7 | EGFR[b] Amplification |
|---|---|---|---|---|---|
| Benign (low grade) (13 cases): | | | | | |
| Oligodendroglioma | 0/3 | 0/3 | 1/2 | 0/3 | 0/3 |
| Astrocytoma | 0/6 | 1/5 | 1/6 | 0/5 | 0/6 |
| Mixed OA | 0/2 | 0/1 | 0/2 | 0/1 | 0/2 |
| Ependymoma | 0/1 | 0/1 | 0/1 | 0/1 | 0/1 |
| Choroid plexus P. | 0/1 | 0/0 | 0/1 | 0/1 | 0/1 |
| Total lost/het. (%) | 0/13 | 1/10 (10) | 2/12 (17) | 0/11 | Total: 0/13 |
| Malignant (high grade) (27 cases): | | | | | |
| Astrocytoma III | 0/5 | 0/4 | 0/5 | 0/4 | 2/5 |
| Oligo-Astro III | 2/2 | 0/2 | 0/2 | 0/0 | 0/2 |
| Glioma, undefined | 0/1 | 0/1 | 0/1 | 0/1 | 0/1 |
| Glioblastoma | 12/19 | 3/18 | 0/19 | 0/18 | 3/16 |
| Total lost/het. (%) | 14/27 (52) | 3/25 (12) | 0/27 | 0/23 | Total: 5/24 (19) |

[a]Number of tumours showing loss of heterozygosity/number of tumours informative (heterozygous) for that probe; [b]Number of tumours showing amplification (>3–5-fold)/number of tumours examined. Chrom., chromosome number. For chromosomes 10 and 17, the result refers to a summary of the results obtained for each chromosome using the various probes and restrictions enzymes listed in Table I. OA and Oligo-Astro – mixed oligodendroglioma-astrocytoma. Het. – heterozygous. P. – papilloma. EGFR – epidermal growth factor receptor.

**Figure 1** Examples of allelic loss at loci on chromosome 10 in tumour number 19, a glioblastoma multiforme. Southern analysis of 10 μg of DNA using the restriction enzymes indicated and the RFLP probes as designated by the HGM symbol. Three different chromosome 10 loci are represented. Panels **a**, **b** and **c** demonstrate loss of heterozygosity in the DNA derived from the tumour (T) when compared to normal DNA derived from the blood (B) of patient 19. In panel **a**, the highest molecular weight band is an invariant (constant) band obtained with the PLAU probe and plays no part in forming the allelic polymorphism (Pearson *et al.*, 1987). Panel **a**, lanes 20 B and T demonstrate an example of maintenance of heterozygosity in a tumour. The allele sizes (A1 and A2) are consistent with those described in Table I, as determined by migration of Lambda phage DNA digested with *Hind*III.

There was no allele loss at the chromosome 1 locus, D1S7, in any of the malignant tumours (all of which were heterozygous for this marker), while two out of 12 (17%) heterozygous benign gliomas showed a loss at this locus. No allele loss was observed at the chromosome 7 locus D7S22 in 11 heterozygous benign tumours, nor in 23 heterozygous malignant tumours.

None of the tumours showed evidence of structural abnormalities of the p53 gene, using the three different restriction enzymes described (data not shown).

*Results of studies on the proto-oncogenes*

Amplification of the EGFR was present in five out of 24 malignant gliomas examined, while none of the 13 benign gliomas showed amplification. Only tumours displaying

**Figure 2** Examples of losses of heterozygosity at loci on chromosome 17. Southern analysis of 10 μg of *Taq*I-digested paired blood (B) and tumour (T) DNA samples probed with the RFLP probe to the D17S30 locus. Loss of heterozygosity in tumour DNA is seen in tumours 21 and 27. Tumour 24 shows maintenance of heterozygosity at this locus.

greater than a 3–5-fold increase in the amount of the EGFR gene, as assessed by the methods used in this study, are listed as having amplification. Two of the tumours with EGFR amplification were malignant astrocytomas, while the remaining three were GBM's (see Table II). In three of the GBM's, the results of the EGFR analysis were not interpretable for technical reasons. Examples of tumours possessing EGFR amplification are shown in Figure 3.

There was no evidence in any of the tumours of amplification of the N-*myc* or of the c-*erb*B-2 proto-oncogenes (data not shown).

Only GBM's possessed multiple molecular abnormalities within the same tumour. Three GBM's had a deletion of loci on chromosome 10 as well as amplification of the EGFR gene; whilst a further GBM had deletions of loci on both chromosomes 10 and 17.

### Discussion

Two major findings emerge from the present study. Firstly, allelic loss of putative TS loci on chromosome 17 were present in both benign and malignant gliomas, whilst loss of loci on chromosome 10 were seen only in malignant gliomas, including GBM's. Secondly, only GBM's showed evidence of loss of loci on more than one chromosome, or both loss of a locus as well as an associated abnormality of an oncogene.

Because of the limitations of the RFLP analysis technique used in this study, the successful demonstration of an allelic

**Figure 3** Amplification of the EGFR gene in malignant gliomas. Southern analysis of 10 μg of *Eco*RI-digested DNA obtained from blood lymphocytes of a healthy volunteer (lane N) and from glioma biopsies, numbers 14 to 16 and 32 to 34. The presence of amplification was assessed by comparing the signal obtained with the p64.1 EGFR probe (panel **A**), with that obtained by the α-2-1 (panel **B**) and α-1-1 (panel **c**) collagen probes. Tumours 15 and 33 show amplification of the EGFR gene. Kbp = molecular weight as assessed by migration of Lambda phage DNA digested with *Hind*III.

loss in a tumour homogenate implies that the majority of the cells in the tumour are members of a single clone possessing that particular genetic abnormality. Loss of an allele indicates loss of part of a chromosome or the entire chromosome. Deletion of material from a specific chromosome in a significant proportion of tumours of a particular histological type is considered to be an indication of a non-random event which may have contributed to the growth of the tumour cells (James *et al*., 1988).

Loss of heterozygosity at one or more of the three loci on chromosome 17 was observed in 10% of informative benign astrocytomas. In the case of malignant tumours, three tumours out of 25 constitutionally heterozygous cases (i.e. 12%) showed loss of an allele. Thus the incidence of deletion of loci on chromosome 17 was similar in both benign and malignant tumours. In contrast, loss of heterozygosity of loci on chromosome 10 was not seen in any of 13 benign constitutionally heterozygous tumours, whereas 14 out of 27 malignant tumours (52%) showed an abnormality. These results concur favourably with the data obtained in studies on non-British patient cohorts, and are consistent with the hypothesis that chromosome 17p loss may represent an event in the development of benign glial tumours, while loss of chromosome 10 alleles is associated with a transition to a malignant phenotype (James *et al*., 1988; James *et al*., 1989; El-Azouzi *et al*., 1989; and Fujimoto *et al*., 1989).

This study failed to identify loss of an allele at the chromosome 7p locus in the tumours. Similarly, the failure of any of the malignant tumours to display loss of the chromosome 1 allele examined suggests that this particular molecular event is not detectable in the majority of the cells in these malignant tumours. Two of the benign tumours did show allelic loss at the chromosome 1 locus, and further studies are needed to determine whether these losses are indicative of a specific and reproducible molecular event which contributes to the development of benign gliomas. The techniques used in the present study may not reveal loss of heterozygosity in a minority of tumour cells, and as many malignant gliomas show pronounced microscopic heterogeneity, it is possible that a subset of the cells making up certain malignant gliomas also have loss of alleles on chromosome 1.

The p53 gene, situated on chromosome 17p, is believed to function normally as a TS gene, and may therefore represent a specific target of abnormalities of chromosome 17 seen here. We therefore examined the p53 gene directly by probing Southern blots with the full-length p53 cDNA probe. This approach failed to reveal any abnormalities of the p53 gene, although it has proved successful in the past in demonstrating p53 abnormalities in osteosarcomas (Masuda *et al*., 1987). It is possible that direct sequencing of the amplified gene product may reveal more subtle abnormalities such as point mutations, similar to those found in colorectal carcinomas (Baker *et al*., 1989).

Only glioblastomas, the most malignant of the gliomas, revealed multiple molecular abnormalities within the same tumour. Thus, three GBM's had a deletion of loci on chromosome 10q, as well as amplification of the EGFR gene. A further GBM had deletions of loci on both chromosome 10 and 17. The presence in the same cell of two or more oncogenes from different functional groups may have a cumulative effect, resulting in the evolution of a more malignant phenotype (Land *et al*., 1983). Similarly, abnormalities of both oncogenes and TS genes occurring in the same cell may result in increased tumourigenicity (Weinberg, 1989). It is therefore possible that the presence in three of the GBM's of loss of putative TS gene loci as well as amplification of the EGFR, a membrane-bound oncogene, may be associated in some way with the development of the highly malignant GBM phenotype. Similarly, as observed in one additional GBM, the association of loss of genetic material from more than one chromosome, which might be compatible with abnormalities of multiple TS loci, may also have a cumulative effect resulting in increased tumourigenicity. Abnormalities of several different oncogenes and/or TS genes may ultimately lead to the same malignant phenotypic end point. Further studies are necessary to establish whether this is the case.

On the basis of the data presented here, it is possible to associate certain molecular abnormalities with gliomas of increasing grades of malignancy. Thus, benign gliomas (grades I and II) may possess abnormalities of putative TS loci on chromosome 17p, as well as on chromosome 1. The development of malignancy (histological grade III) is frequently associated with loss of a locus on chromosome 10, or of the whole chromosome. The presence of an associated abnormality of an oncogene such as amplification of the EGFR gene, is associated with the development of an even more malignant phenotype, the GBM's. The association of stepwise genetic lesions with increasing grades of glial malignancy may provide the basis for a testable model of the molecular events underlying glial tumour progression, similar to the model proposed for colorectal tumours (Vogelstein *et al*., 1988).

The association of specific molecular genetic lesions with differing grades of malignancy may also prove of value in tumour diagnosis. The choice of treatment given to a particular patient is often based on the histological grading of the tumour. Given the small size of many of the biopsies submitted for diagnosis, coupled with the extreme morphologic heterogeneity present in some gliomas, it may be impossible to identify a minority of malignant cells in an otherwise benign tumour. However, this small proportion of malignant cells may represent a subclone which may ultimately determine the biological behaviour of the glioma. Such malignant subclones may be identifiable in the future by the use of gene amplification techniques, coupled with knowledge of the molecular genetic lesions associated with specific stages of glial tumour progression.

We thank Drs W. Cavenee, Y. Nakamura, R. White, M. Carlson, M. Litt, H. McDermid, G. Sebastio, T. Dryja, P. Raeymaekers, T. Yamamoto, J. Jenkins, C. Mathew and Cellmark Diagnostics (courtesy Professor A.J. Jeffreys), for providing the probes used in this study. Drs W. Cavenee and C. Mathew provided support and helpful advice. Professor L. Duchen reviewed many of the histological sections.

**Note added in proof.**

Further analysis on the same cohort of tumours revealed a deletion of exons 18–24 of the Retinoblastoma gene (RB) in one of the GBM's. Three further GBM's showed loss of heterozygosity at an RFLP locus situated within the RB gene. Thus, 44% of the heterozygous GBM's showed deletions within the RB gene. The RB gene deletions were not present in gliomas of lower malignancy grade. Deletions within the RB gene may therefore constitute a further example of a sequential molecular abnormality confined to a specific stage of human glioma evolution (Venter, *et al.*, 1991).

### References

BAKER, S.J., FEARON, E.R., NIGRO & 9 others (1989). Chromosome 17 deletions and p53 gene mutations in colorectal carcinomas. *Science*, **244**, 217.

BIGNER, S.H., MARK, J., BULLARD, D.E., MAHALEY, M.S. & BIGNER, D.D. (1986). Chromosomal evolution in malignant human gliomas starts with specific and usually numerical deviations. *Cancer Genet. Cytogenet.*, **22**, 121.

BISHOP, J.M. (1983). Cellular oncogenes and retroviruses. *Ann. Rev. Biochem.*, **52**, 301.

CARLSON, M., NAKAMURA, Y., PAYSON, R. & 5 others (1988). Isolation and mapping of a polymorphic DNA seqence pMCT35.1 on chromosome 17p (D17S31). *Nucleic Acids Res.*, **16**, 783.

CAVENEE, W.K., DRYJA, T.P., PHILLIPS, R.A. & 6 others (1983). Expression of recessive alleles by chromosomal mechanisms in retinoblastoma. *Nature*, **305**, 779.

COOPER, J.A. & WHYTE, P. (1989). RB and the cell cycle: entrance or exit? *Cell*, **58**, 1009.

EL-AZOUZI, M., CHUNG, R.Y., FARMER, G.E. & 10 others (1989). Loss of distinct regions on the short arm of chromosome 17 associated with tumourigenesis of human astrocytomas. *Proc. Natl Acad. Sci. USA*, **86**, 7186.

FEINBERG, A. & VOGELSTEIN, B. (1983). A technique for radiolabelling DNA restriction endonuclease fragments to high specific activity. *Anal. Biochem.*, **132**, 6.

FINLAY, C.A., HINDS, P.W. & LEVINE, A.J. (1989). The p53 proto-oncogene can act as a suppressor of transformation. *Cell*, **57**, 1083.

FUJIMOTO, M., FULTS, D.W., THOMAS, G.A. & 7 others (1989). Loss of heterozygosity on chromosome 10 in human glioblastoma multiforme. *Genomics*, **4**, 210.

GARSON, J.A., MCINTYRE, P.G. & KEMSHEAD, J.T. (1985). N-*myc* amplification in malignant astrocytoma. *Lancet*, **ii**, 718.

HEIM, S. & MITELMAN, F. (1988). *Cancer Cytogenetics*. Alan R. Liss Inc.: New York.

JAMES, C.D., CARLBLOM, E., DUMANSKI, J.P. & 4 others (1988). Clonal genomic alterations in glioma malignancy stages. *Cancer Res.*, **48**, 5546.

JAMES, C.D., CARLBLOM, E., NORDENSKJOLD, M., COLLINS, V.P. & CAVENEE, W.K. (1989). Mitotic recombination of chromosome 17 in astrocytomas. *Proc. Natl Acad. Sci. USA*, **86**, 2858.

KINZLER, K.W., BIGNER, S.H., BIGNER, D.D. & 5 others (1987). Identification of an amplified, highly expressed gene in a human glioma. *Science*, **236**, 70.

KNUDSON, A.G. (1971). Mutation and cancer: statistical study of retinoblastoma. *Proc. Natl Acad. Sci. USA*, **68**, 820.

LAMB, P. & CRAWFORD, L. (1985). Characterisation of the human p53 gene. *Mol. Cell Biol.*, **6**, 1379.

LAND, H., PARADA, L.F. & WEINBERG, R.A. (1983). Cellular oncogenes and multistep carcinogenesis. *Science*, **222**, 771.

LIBERMANN, T.A., NUSBAUM, H.R., RAZON, N. & 7 others (1985). Amplification, enhanced expression and possible rearrangement of EGF receptor gene in primary human brain tumours of glial origin. *Nature*, **313**, 144.

LITT, M., MUELLER, O.T., SHOWS, T.B. & LITT, R. (1987). A single copy subclone, p1-101, from cosmid 3-3B, defines three RFLP's on 10pter-q23 (HGM9 no. D10S4). *Nucleic Acids Res.*, **15**, 2783.

MASUDA, H., MILLER, C., KOEFFLER, H.P., BATTIFORA, H.J. & CLINE, M.J. (1987). Rearrangement of the p53 gene in human osteogenic sarcomas. *Proc. Natl Acad. Sci. USA*, **84**, 7716.

MCCOMB, R.D. & BIGNER, D.D. (1984). The biology of malignant gliomas – a comprehensive survey. *Clin. Neuropathol.*, **3**, 93.

MCDERMID, H.E., GOODFELLOW, P.J., DUNCAN, A.M.V. & 5 others (1987). A polymorphic locus, D10S5, at 10q21.1. *Nucleic Acids Res.*, **15**, 5498.

NAKAMURA, Y., LEPPERT, M., O'CONNELL, P. & 8 others (1987). Variable number of tandem repeat (VNTR) markers for human gene mapping. *Science*, **235**, 1616.

PEARSON, P.L., KIDD, K.K. & WILLARD, H.F. (1987). Human gene mapping by recombinant DNA techniques. *Cytogenet. Cell Genet.*, **46**, 390.

RAEYMAEKERS, P., VAN BROECKHOVEN, C. & VANDENBERGHE, A. (1988). Two polymorphic loci are detected simultaneously by probe CARLP 118.2 (D10S21), on chromosome 10. *Nucleic Acids Res.*, **16**, 2738.

REY, J.A., BELLO, M.J., DECAMPOS, J.M., KUSAK, M.E. & MORENO, S. (1987). Chromosomal composition of a series of 22 human low-grade gliomas. *Cancer Genet. Cytogenet.*, **29**, 223.

SCHECHTER, A.L., STERN, D.F., VAIDYANATHAN, L. & 4 others (1984). The neu oncogene: an erb-B-related gene encoding a 185,000-Mr tumour antigen. *Nature*, **312**, 513.

SCHWAB, M., ALITALO, K., KLEMPNAUER, K.-H., VARMUS, H.E., BISHOP, J.M., GILBERT, F., BRODEUR, G., GOLDSTEIN, M. & TRENT, J. (1983). Amplified DNA with limited homology to myc cellular oncogene is shared by human neuroblastoma cell lines and a neuroblastoma tumour. *Nature*, **305**, 245.

SEBASTIO, G., RICCIO, A., VERDE, P., SCARPATO, N. & BLASI, F. (1985). BamHi RFLP linked to the human urokinase gene. *Nucleic Acids Res.*, **13**, 5404.

SLAMON, D.J., GODOLPHIN, W., JONES, L.A. & 8 others (1989). Studies of the HER-2/neu proto-oncogene in human breast and ovarian cancer. *Science*, **244**, 707.

SOLOMON, E., HIORNS, L., SHEER, D. & ROWE, D. (1984). Confirmation that the Type I collagen gene on chromosome 17 is COL1A1 (x-1(I)), using a human genomic probe. *Ann. Human Genet.*, **48**, 39.

SYKES, B. & SOLOMON, E. (1978). Assignment of a type I collagen structural gene to human chromosome 7. *Nature*, **272**, 548.

T'ANG, A., VARLEY, J.M., CHAKRABORTY, S., MURPHREE, A.L. & FUNG, Y.-K.T. (1988). Structural rearrangement of the retinoblastoma gene in human breast carcinoma. *Science*, **242**, 263.

ULLRICH, A., COUSSENS, L., HAYFLICK, J.S. & 12 others (1984). Human epidermal growth factor receptor cDNA sequence and aberrant expression of the amplified gene in A431 epidermoid carcinoma cells. *Nature*, **309**, 418.

VENTER, D.J., TUZI, N.L., KUMAR, S. & GULLICK, W.J. (1987). Overexpression of the c-*erb*B-2 oncoprotein in human breast carcinomas: immunohistological assessment correlates with gene amplification. *Lancet*, **ii**, 69.

VOGELSTEIN, B., FEARON, E.R., HAMILTON, S.R. & 7 others (1988). Genetic alterations during colorectal-tumour development. *N. Engl. J. Med.*, **319**, 525–532.

WEINBERG, R.A. (1989). Oncogenes, antioncogenes, and the molecular basis of multistep carcinogenesis. *Cancer Res.*, **49**, 3713.

WONG, Z., WILSON, V., PATEL, I., POVEY, S. & JEFFRIES, A.J. (1987). Characterisation of a panel of highly variable minisatellites cloned from human DNA. *Ann. Hum. Genet.*, **51**, 269.

YOKOTA, J., YAMAMOTO, T., YOYOSHIMA, K. & 4 others (1986). Amplification of the c-*erb*B-2 oncogene in human adenocarcinomas *in vivo*. *Lancet*, **i**, 765.